# Data on RNA-seq analysis of *Garcinia mangostana* L. seed development

**DOI:** 10.1016/j.dib.2017.11.001

**Published:** 2017-11-07

**Authors:** Othman Mazlan, Azhani Abdul-Rahman, Hoe-Han Goh, Wan Mohd Aizat, Normah Mohd Noor

**Affiliations:** Institute of Systems Biology (INBIOSIS), Universiti Kebangsaan Malaysia (UKM), 43600 Bangi, Selangor, Malaysia

**Keywords:** Mangosteen fruit, Seed development, Transcriptomics, RNA sequencing

## Abstract

Mangosteen (*Garcinia mangostana* L.) has exceptional potential for commercial and pharmaceutical applications due to its delicious fruit and medicinal properties. Nevertheless, the molecular mechanism of mangosteen seed development is poorly understood. In this study, we performed transcriptomic analysis of four seed developmental stages; eight, ten, twelve and fourteen weeks after anthesis. Illumina HiSeq™ 4000 sequencer was used to generate raw data of approximately 68 Gb in size. From 451,495,326 raw reads, 406,143,756 clean reads were obtained. The raw data were uploaded to SRA database and the BioProject ID is PRJNA395504. These data provide the basis for further exploration and understanding of the molecular mechanism in mangosteen seed development.

**Specifications Table**

**Table:** 

Subject area	Biology
More specific subject area	Transcriptomics
Type of data	Tables, text file
How data was acquired	Illumina HiSeq™ 4000
Data format	Raw (FASTQ)
Experimental factors	Mangosteen seed development; eight, ten, twelve and fourteen weeks after anthesis.
Experimental features	Transcriptome of mangosteen seed development
Data source location	UKM Bangi, Malaysia (2°55′09.0″N 101°47′04.8″E)
Data accessibility	Data can be accessed from NCBI SRA (BioProject ID: PRJNA395504) (https://www.ncbi.nlm.nih.gov/bioproject/PRJNA395504)

**Value of the data**

• The data obtained using Illumina sequencer is the first report on RNA-seq of mangosteen seed at different developmental stages (eight, ten, twelve and fourteen weeks after anthesis).
• This permits the identification of differentially expressed genes that may play an important role in mangosteen seed development.
• Transcriptomics analysis provides the foundation in elucidating the molecular regulation during mangosteen seed development. Data obtained will be valuable for further investigation on putative genes and proteins discovery in mangosteen seed development.

## 1 Data

This dataset are raw reads for mangosteen seed at four different developmental stages; eight, ten, twelve and fourteen weeks after anthesis. Consequently, the data were *de novo* assembled into full-length transcriptome.

## 2 Experimental design, materials and methods

### 2.1 Plant materials

Mangosteen fruit were obtained from mangosteen plots at Universiti Kebangsaan Malaysia, Bangi (2°55′09.0″N 101°47′04.8″E). Flowers of mangosteen were labelled at anthesis during its flowering season (March – April 2014). During fruiting period (June – August 2014), fruits were harvested for seeds at eight, ten, twelve and fourteen weeks after anthesis denoting different developmental stages. Seed samples were stored at −80 °C and grounded to fine powder prior analysis.

### 2.2 Total RNA extraction and quality control, library preparation and transcriptomic service

Extraction of seed total RNA was done [1], [2] via modifying the CTAB method [3]. For quality control, NanoDrop spectrophotometer (Thermo Fisher Scientific Inc., USA) and Agilent 2100 Bioanalyzer (Agilent Technologies, USA) were used to determine the total RNA quantity, quality and reliability. Samples with RNA integrity number (RIN) of around 8.0 or higher were selected for library preparation and sequencing. The mRNA library preparation employed was SureSelect Strand-Specific RNA Library Prep for Illumina Multiplexed Sequencing (protocol version E0, March 2017). Consequently, RNA-seq was performed using Illumina HiSeq™ 4000 (Theragene Etex, South Korea), generating 150 bp of paired end reads (Table 1).

**Table 1:** SRA accession numbers and links for raw data of mangosteen seed development transcriptome. Mangosteen seed developmental stages; eight (W8), ten (W10), twelve (12) and fourteen (W14) weeks after anthesis.

**Stage**	**Replicate**	**Accession number**	**Accession links**
W8	GmW8-A	SRX3066480	http://www.ncbi.nlm.nih.gov/sra/SRX3066480
	GmW8-B	SRX3066479	http://www.ncbi.nlm.nih.gov/sra/SRX3066479
W10	GmW10-A	SRX3066478	http://www.ncbi.nlm.nih.gov/sra/SRX3066478
	GmW10-B	SRX3066477	http://www.ncbi.nlm.nih.gov/sra/SRX3066477
W12	GmW12-A	SRX3066481	http://www.ncbi.nlm.nih.gov/sra/SRX3066481
	GmW12-B	SRX3066475	http://www.ncbi.nlm.nih.gov/sra/SRX3066475
W14	GmW14-A	SRX3066474	http://www.ncbi.nlm.nih.gov/sra/SRX3066474
	GmW14-B	SRX3066476	http://www.ncbi.nlm.nih.gov/sra/SRX3066476

### 2.3 *De novo* transcriptome assembly

Quality control of raw reads were tested via FastQC version 0.11.2 [4]. Then, high quality reads were obtained by trimming adapters and other unwanted sequences sequence using cutadapt version 1.9.1 [5] and filtering the reads using in-house script by Theragen Etex Bio Institute, Republic of Korea (Table 2). Trinity version 2.1.1 [6] was used to assemble the reads *de novo* [7] with default configuration while TIGR Gene Indices clustering tools version 2.1 (Identity; 0.94) [8] was used to omit redundant sequences and cluster them into non-redundant unigenes set. A total of 101,384 unigenes were found and their average length is 784 bp (Table 3).

**Table 2:** Statistics of raw and clean reads and bases of mangosteen seed development transcriptome. Mangosteen seed developmental stages; eight (W8), ten (W10), twelve (12) and fourteen (W14) weeks after anthesis.

**Stage**	**Replicate**	**Raw**		**Clean**	
		**Reads**	**Bases (bp)**	**Reads**	**Bases (bp)**
W8	GmW8-A	61,372,766	9,267,287,666	58,496,864	7,896,242,284
	GmW8-B	57,393,260	8,666,382,260	54,766,226	7,410,657,947
W10	GmW10-A	55,824,356	8,429,477,756	53,424,608	7,198,009,302
	GmW10-B	47,977,546	7,244,609,446	45,998,728	6,149,885,945
W12	GmW12-A	52,078,760	7,863,892,760	31,555,052	4,113,212,390
	GmW12-B	49,468,874	7,469,799,974	46,959,954	6,170,160,791
W14	GmW14-A	64,984,566	9,812,669,466	62,069,538	7,964,059,718
	GmW14-B	62,395,198	9,421,674,898	52,872,786	6,933,874,925

**Table 3:** Statistics of mangosteen seed development transcriptome assembly.

**Attributes**	**Value**
**Pre-assembly**	
Total raw reads (bases, bp)	451,495,326 (68,175,794,226 bp)
Total clean reads (bases, bp)	406,143,756 (53,836,103,302 bp)
**Post-assembly**	
Number of unigenes (bases, bp)	101,384 (79,466,965 bp)
Average length of unigenes	784 bp
N50	–

## References

[1] Abdul-Rahman A., Suleman N.I., Wan Zakaria W.N.A., Goh H.H., Aizat W.M. (2017). RNA extraction of mangosteen (*Garcinia**mangostana* L.) pericarps for RNA sequencing. Sains Malays..

[2] Abdul-Rahman A., Goh H.-H., Loke K.-K., Noor N.M., Aizat W.M. (2017). RNA-seq analysis of mangosteen (*Garcinia**mangostana* L.) fruit ripening. Genom. Data.

[3] Kim S.H., Hamada T. (2005). Rapid and reliable method of extracting DNA and RNA from sweetpotato, *Ipomoea batatas* (L). Lam. Biotechnol. Lett..

[4] S. Andrews, FastQC A Quality Control tool for High Throughput Sequence Data, 2010 〈http://www.bioinformatics.babraham.ac.uk/projects/fastqc/〉.

[5] Martin M. (2011). Cutadapt removes adapter sequences from high-throughput sequencing reads. EMBnet J..

[6] Grabherr M.G., Haas B.J., Yassour M., Levin J.Z., Thompson D.A., Amit I., Adiconis X., Fan L., Raychowdhury R., Zeng Q., Chen Z., Mauceli E., Hacohen N., Gnirke A., Rhind N., di Palma F., Birren B.W., Nusbaum C., Lindblad-Toh K., Friedman N., Regev A. (2011). Trinity: reconstructing a full-length transcriptome without a genome from RNA-Seq data. Nat. Biotechnol..

[7] Haas B.J., Papanicolaou A., Yassour M., Grabherr M., Blood P.D., Bowden J., Couger M.B., Eccles D., Li B., Lieber M., MacManes M.D., Ott M., Orvis J., Pochet N., Strozzi F., Weeks N., Westerman R., William T., Dewey C.N., Henschel R., LeDuc R.D., Friedman N., Regev A. (2013). De novo transcript sequence reconstruction from RNA-seq using the Trinity platform for reference generation and analysis. Nat. Protoc..

[8] Pertea G., Huang X., Liang F., Antonescu V., Sultana R., Karamycheva S., Lee Y., White J., Cheung F., Parvizi B., Tsai J., Quackenbush J. (2003). TIGR Gene Indices clustering tools (TGICL): a software system for fast clustering of large EST datasets. Bioinformatics.

